# Pilot testing the Engaging Generations (eGen) Program to address social well-being among lower-income older adults

**DOI:** 10.3389/fpubh.2024.1341713

**Published:** 2024-08-09

**Authors:** Cindy E. Tsotsoros, Emma Pascuzzi, Melanie Brasher, Kristin Souza, Skye N. Leedahl

**Affiliations:** ^1^Department of Human Development and Family Science, The University of Rhode Island, Kingston, RI, United States; ^2^George & Anne Ryan Institute for Neuroscience, The University of Rhode Island, Kingston, RI, United States; ^3^Department of Sociology and Anthropology, The University of Rhode Island, Kingston, RI, United States; ^4^Center for Career and Experiential Education, The University of Rhode Island, Kingston, RI, United States

**Keywords:** intergenerational relationships, technology, social isolation, loneliness, quality of life, intervention

## Abstract

**Introduction:**

Throughout the COVID-19 pandemic, the need to address digital inclusion and social well-being for older adults was particularly apparent for those from disadvantaged communities. This pilot program provided access to technology and intergenerational mentorship to older adult participants interested in receiving and learning how to utilize an iPad. Pre/post-changes were examined for social well-being in the areas of quality of life, social isolation, and loneliness.

**Methods:**

This study conducted pre- and post-surveys with older participants (*n* = 145) from five disadvantaged communities in the United States utilizing standardized measures. One-on-one interviews were conducted post-program (*n* = 98) to examine participants’ perceptions of the program and evaluate its impact on social measures.

**Results:**

The study sample included older adults (Mean age = 72.3) who were mostly lower income (82.3%) and self-reported as Black (13.6%), Hispanic (21.7%), and White (56.5%). Significant differences were identified in participant pre/post-survey scores for social isolation, loneliness, and a global measure of quality of life. Qualitative analyses suggest improvements in various aspects of social well-being. Themes showed that participants believed the program contributed to (1) enhanced mood and mental health, (2) improved quality of life, (3) sense of purpose and feelings of being less alone, (4) ability to use video calling to connect with others; and (5) ability to more freely use email, texting, and messaging to communicate with others.

**Discussion:**

This research demonstrates that this pilot program seemed to contribute to reduced social isolation and loneliness for participants, and participants stated more positive social well-being following program participation. However, future research with larger samples is needed to expand upon these findings. Future studies will examine the pathways between technology improvements and social well-being and examine group differences.

## 1 Introduction

The COVID-19 social connectivity paradox posited that older adults reduced social interactions to protect against COVID-19 and other illnesses but increased their risk for social isolation (SI), loneliness, and reduced quality of life (QOL) (1). The risk was heightened for individuals already experiencing SI or disconnectedness prior to the pandemic (1–4), and the ramifications of this paradox “will be seen for months and years to come (1).” The convergence of the COVID-19 social connectivity paradox and the recognized digital divide for older adults (5) motivated programs across the country toward innovation to meet community needs during the height of the pandemic (6–8). Furthermore, the need to address social well-being issues and enhance digital inclusion was particularly apparent for older adults from disadvantaged communities, such as those with lower income, with disabilities, and who do not speak English (1, 4).

QOL is defined as the degree to which an individual is healthy, comfortable, and able to enjoy and participate in life experiences. Older adults tend to have a lower QOL due to geriatric syndromes such as lower cognition, depressive symptoms, functional limitations, and additional chronic illnesses (9–12). The effects of the COVID-19 quarantine significantly impacted older adults and their QOL. This was especially true during “lockdown,” the implementation of stay-at-home orders, curfews, quarantines, and societal restrictions. Individuals older than 50 in Chile felt sad or depressed during the lockdown, with confinement increasing anxiety and depressive symptoms (12). A longitudinal study (13) followed an older adult population throughout the two lockdown periods in Canada and examined QOL. Results suggested that QOL was reduced during the pandemic and linked to physical activity, energy, happiness, and perceived isolation.

Social isolation (SI) is associated with QOL but is a distinct aspect of social well-being and is defined as an individual’s physical and/or psychological distancing from their networks of desired or needed relationships with others (14). Research has found SI to be a risk factor for poorer physical and mental health (15), including an increased risk of developing Alzheimer’s disease (16), higher mortality risk specifically for Dutch males (17), and reduced cognitive functioning (18). Prior to the pandemic, although many older adults were active participants in social activities, such as community events, attending senior centers, church events, and travel (19); worries about SI were still a common trend. A qualitative study of 30 older adults in Canada revealed that half the participants brought up themes of exclusion (20). Throughout the world, aging and isolation during the pandemic negatively impacted older adults’ emotional well-being, making them easily frustrated and feeling helpless (21).

SI is a known risk factor for experiencing loneliness (22). Loneliness, another multi-faceted aspect of social well-being, is an unpleasant and unwelcome feeling (23) and a painful feeling that occurs when one is not as socially or intimately connected to people in their network, as desired (24). Individuals with lower socioeconomic status and those with poor-quality relationships are at an increased risk of emotional loneliness (25). Unsurprisingly, individuals of all ages experienced increased loneliness at the beginning of the pandemic. Specifically, the older adult population (26) found themselves isolated to reduce COVID-19 health risks. In June 2020, more than half of older adults (56%) in the US reported feeling isolated from others compared to 27% in 2018. A New Zealand study found that loneliness in older adults is significantly associated with depression and suicidal ideation, particularly for minority groups and females (26).

Technology-based interventions have been used to address social well-being in older age by examining outcomes such as SI, loneliness, and social connectedness (27) with most showing little evidence of effectiveness (28). Researchers found that a specially designed computer system for personal reminders and social management assistance effectively reduces loneliness in older adults (29). However, reducing SI was not identified in significance testing. Prior to the pandemic, complex, multi-strategy and technology-related interventions showed the most promise for reducing SI and/or loneliness, but it was recognized that the literature in this area was vast and in need of measurement refinement and more conclusive findings (30). In reviewing technology-based interventions (31), most interventions showed positive but somewhat varied results in reducing social isolation and loneliness among older adults with video games, PRISM, tele-care, general information and communication technologies, and robotics showing promising but not robust findings. A 2020 review (28) found that internet access was fundamental in supporting long-distance interactions, that most interventions provide training and support, and that different combinations of technologies, such as video chat, email, and social networks, were favored as technology-related interventions for improving social well-being in older age. However, a study of older adults from 21 different countries during the COVID-19 pandemic identified that dissatisfaction with video calling contributed to feelings of loneliness and increased isolation (32). Finally, a systematic review (33) regarding older adults in Australia and the United States indicated the growth in popularity of touch-screen technology usage among older adults. This is due to the ease in which older adults can engage in features that promote social interaction, such as sharing photographs or initiating video conferences.

In response to the feelings of loneliness caused by the pandemic, some older adults were motivated to begin learning and expanding their knowledge and use of social technology to stay connected with their friends and family (34). Unfortunately, those without knowledge or access to technology were unable to utilize this form of communication. The disadvantage for older adults who lacked technological skills became more apparent during the COVID-19 lockdown, as the Centers for Disease Control and Prevention (CDC) (35) recommended that families communicate with their loved ones in long-term care centers or those immunocompromised via technology. Failing to provide a recommendation for older adults who lacked technological skills left the needs of those still suffering from SI unaddressed. A prior systematic review (28) highlighted that while there is no evidence that technology-based interventions cause any harm, they might amplify feelings of SI among participants who lack the necessary physical or mental capabilities, or those lacking confidence in technology usage. Furthermore, the same review found a diverse range of interventions with no defined key elements consistent across groups or types of loneliness but that tailoring the intervention to the specific needs of individuals would improve the results. There is a particular need to identify evidence-based interventions for addressing social well-being among low-income older adults of diverse racial and ethnic backgrounds, with one study in San Francisco showing evidence of success with a peer program involving home visits and community connections (36). Ensuring older adults from Spanish-speaking communities are included in outreach efforts and educational interventions is also suggested since evidence shows strong connections between language segregation and depressive symptoms among older Latinos (37).

Since the late 1970s, intergenerational programs have been implemented in educational settings to bridge the divide between older and younger generations, allowing these individuals to nurture and support each other (38). These programs have allowed older generations to pass along wisdom, values, and life experiences to younger generations (39), and much of the research on intergenerational programs has focused on challenging young adults’ stereotypes of older adults (40). Some exceptions to this trend include programs focused on the needs of older adults, including social needs (41), reduced negative self-perceptions and depression (42), and well-being (43). Researchers emphasize how building friendships, providing training, mentoring, using technology, and promoting cooperation are evidence-based intergenerational practices (44). An interprofessional pilot study utilizing an intergenerational program to combat loneliness and isolation among older adults identified, from the student perspective, positive social interaction benefits for older adults and students (8); however, data were not collected from older adults. Recent research has shown that loneliness for older adults can be influenced by intergenerational technology programs (45).

While many programs across the country found new ways to get technology into the hands of older adults during the pandemic (7), not many collected data, leaving researchers unable to rigorously examine connections between technology use and social well-being. Based on the research, we believe that intergenerational technology programs may help decrease SI and loneliness and improve QOL in older adults by allowing them to adapt and learn new technologies, partake in social activities and connect with others. This study sought to determine if a program combining intergenerational and technological elements could better address social well-being for older adults.

This study is guided by social exchange theory that emphasizes how relationships (older and younger) are often focused on avoiding costs/difficulties and pursuing rewards/benefits (46) and contact theory that addresses the value of building trust and confidence across generations (47). Regarding older adult learning, the Knowles theory of andragogy (48) and sociocultural learning theory (49) guided program development. Last, this pilot aligns with many of the tenets of the implementation science framework (50), which emphasizes how the maximal benefit of a program/intervention is best realized through ongoing development, evaluation, and refinement within diverse populations and systems and that sustainability/success can happen when there is a reciprocal fit within a practice setting and the larger ecological system.

The Cyber-Seniors Organization (51) offers an intergenerational technology program that bridges the digital divide by training younger persons to assist older adults in technological learning. As one of the partners, The University of Rhode Island Engaging Generations (URI eGen) Program successfully created intergenerational infrastructure with university/community partnerships to help older adults digitally connect with others (52) and found improvements in technology use and digital competence among older adults (53). However, the outcomes related to social well-being have varied, and prior to the pandemic, the samples lacked economic or racial/ethnic diversity or included already experienced technology users (52–54).

When the COVID-19 pandemic began, the eGen Program greatly expanded its efforts and received funding for a pilot from the state unit on aging to enhance digital inclusion for older adults from disadvantaged communities, alleviate SI in the at-risk older adult population, and combat COVID-exacerbated ageism (55). This goal was met by offering an intergenerational program, developed using previous experience and the literature, to support older adults’ continued learning, growth, and meaningful connections. In eGen, both generations benefit, with older participants learning technology to improve their lives and younger participants gaining professional experience/internship/service hours while building trust and confidence through multiple interactions focused on growth and development. The idea is that this reciprocity across generations helps everyone learn from and about those with divergent perspectives from their own. In eGen, there is a strong fit between the program and the implementation setting (senior centers), and we are continuously focused on utilizing evaluative research to refine systems and tailor the program to meet needs.

This study conducted an intervention within community/senior centers focused on increasing technology access (i.e., providing an iPad and internet connection). We utilized an intergenerational approach to help older adults inexperienced with technology to learn the basics of using the iPad and utilize apps or programs available to enrich their lives in a person-centered way to enhance their social well-being. This combination of features was designed based on previous literature and experience and offered a novel contribution to the literature compared to previous programs/interventions. The data came from a larger study examining technological outcomes, which showed improvements in older participants’ technology use and digital competence (54). Future work will evaluate outcomes for younger participants. Although many studies have inferred that greater online use can serve as a tool to enhance social connectedness, these studies fall short in identifying how technology programs can improve community engagement among older adults.

The current study aims to address gaps in the literature by piloting an intergenerational technology program to address social well-being in older adults. This pilot utilizes multiple social well-being measures from a diverse sample of technologically inexperienced older adults. The novel contribution to the literature is that URI eGen differentiates itself from previous interventions, incorporating participation across the state and gathering insight on QOL, SI, and loneliness from older adults of various demographic and socioeconomic backgrounds.

As part of implementing URI eGen, two research questions guided the methodology for this mixed methods research:

1. Were significant improvements detected in quality of life, social isolation, and loneliness from pre- to post-survey for older participants?
2. How did the pilot contribute to social well-being from the perspective of the participants?

The hypotheses were that individuals who participated in the pilot program would show improvements in QOL, SI, and loneliness.

## 2 Materials and methods

### 2.1 Research design

These data were collected using a mixed methods convergent parallel design; quantitative and qualitative data were collected simultaneously, analyzed separately, and the findings were compared to draw overall conclusions (56). This study was approved by the university IRB (769500).

#### 2.1.1 Recruitment

The inclusion criteria for older adult participants were: (1) aged 50 or older; (2) residence in the five selected communities; (3) lack of and desire a digital device &/or internet access; (4) English or Spanish-speaking; (5) willingness to receive 3 months of technology training with student mentors; (6) willingness to take part in the research study. eGen worked with the state unit on aging to determine the age-cut off based on identified needs within the state, such as workforce/job retraining purposes as well as health/social needs. The five geographically dispersed senior/community centers in mostly urban areas were chosen to participate in the study due to having higher COVID-19 rates at the time (2021) and due to being ideal spaces for participant recruitment. These five sites included four senior centers dedicated to older adult life enrichment for those living in the community, and one was a community center with a dedicated senior program for community-dwelling older adults. These sites were located in communities with higher proportions of lower-income populations that were racially/ethnically diverse (English- and Spanish-speaking) to accomplish our goal of promoting social and economic equity. Recruitment was by printed flyers and emailed newsletters. Interested individuals called the centers, and staff members sent registration information to the study team. These efforts resulted in 272 people showing interest in the pilot study.

#### 2.1.2 Data collection

After participants provided informed consent, students asked pre-survey questions over the phone and entered data electronically. Of the 272 people who registered, there were 184 participants who completed the pre-survey questions and received an iPad, thus becoming part of the study sample in 2021. Once participants completed eGen, they completed the post-survey (phone) and were told they could keep the iPad (incentive for study completion). In cases where a participant did not complete the program during their time with the student (*n* = 46), they were re-assigned the following semester and given the post-survey after completion (completion generally occurred within 2 months). Some of those participants are not included in post-survey analyses, as the data was not available at the end of 2021 (*n* = 24); the remaining never finished the program. Similar to the pre-survey, the post-survey also included program evaluation questions. Student researchers asked participants if they would participate in a short, audio-recorded interview about their experiences. There were 145 participants who completed the post-survey questionnaire (78.8% completion/response rate), and of those, 98 agreed to participate in the qualitative interview. The researchers informed participants that this was their chance to give details about eGen and how it may have influenced their lives. If the participant agreed, the researcher began recording (recordings were professionally transcribed). Recordings in Spanish were transcribed in Spanish, translated into English (translation service), and verified by bilingual student researchers. Transcripts were uploaded into qualitative software, NVivo, for analysis (57).

#### 2.1.3 Intervention elements

Participants completed an over-the-phone pre-survey, then were given a new iPad with Wi-Fi capability (hotspot device with unlimited data given to those without internet), binder kit, screen protector, iPad cover, and styluses. The iPads were pre-loaded with various applications and links to state resources. In addition to many of the standard Apple apps, such as iMessage and FaceTime, the additional preloaded apps included Zoom, Facebook, Instagram, YouTube, Spotify, Pandora, and Talkatone. The binders (available in English and Spanish) included program information, resources, and instructions/suggestions designed for older adults. Participants were assigned student mentors whom they met with for about an hour weekly or biweekly (ideally about 4–5 times throughout the semester over a 4-month period) via phone or Zoom, though this did vary based on individual interest and availability (*M* = 3.5, range = 1–24). The number of meetings was intentionally individualized to meet each person’s needs. During these meetings, the intergenerational pairs worked toward meeting learning goals from the program checklist, and each person varied in how quickly they learned the items included on the checklist. Additional optional group meetings were offered to participants to discuss technology-related topics. For a more detailed description of the eGen pilot program, please see *Pilot Program Elements* (54).

### 2.2 Measures

#### 2.2.1 Quality of life

The Older People’s Quality of Life questionnaire [(58); OPQOL] was used to measure quality of life. The scale contains 13 items with responses on a 5-point Likert scale, ranging from 1 *(strongly disagree)* to 5 *(strongly agree)*. A composite score is constructed by summing the 13 responses (higher scores indicate better quality of life). The alpha for the pre-and post-survey were 0.854 and 0.922, respectively. The quality-of-life scale also includes a global question that asks respondents to rate their overall quality of life from 1 *(very good)* to 5 *(very bad)*. For the global question, lower scores indicate better quality of life.

#### 2.2.2 Social isolation

The scale used to measure SI was the Social Isolation Scale (14). This scale contains six questions examining interactions with others, relationships, and group belonging. Three items pertain to frequency of interactions, with response options being *none,* 1, 2–3, 4–5, 6 *or more*. Three questions ask about relationships with individuals or groups. Respondents are asked to which level they agree, 1 *(strongly disagree)* to 5 *(strongly agree).* The composite score is summed responses, with lower scores indicating more isolation. The six questions are separated into two subscales. The subscales examine connectedness and belongingness. Within these subscales, scores can range from 3 to 15. The calculated McDonald’s omega was 0.701 for the pre-survey and 0.746 for the post-survey.

#### 2.2.3 Loneliness

The Loneliness Scale (59) was used to measure feelings of loneliness. The scale contains six items with response options of *Yes* (1), *More or less* (0), and *No* (0). Composite scores are summed responses, with higher scores indicating more feelings of loneliness. The calculated McDonald’s omega was 0.727 for the pre-survey scale and 0.680 for the post-survey scale. Two subscales are empirically validated, Emotional Loneliness and Social Loneliness, with each factor containing three questions with a range of 0–3.

#### 2.2.4 Demographics

Demographic variables were collected pre-survey, including age, gender, race and ethnicity, primary language, relationship status, employment status, living arrangements, annual household income, highest level of education, and self-reported health status.

#### 2.2.5 Interview

Open-ended interview questions included the following: What was your favorite part of the program? What has it meant for you to be involved in the program? Has your iPad helped you connect with family and friends in different ways? What social groups or activities have you joined (or been able to do) since getting your iPad?

### 2.3 Analysis

To address aim one, items and scales were analyzed from the pre- and post-surveys. Changes in score from pre- to post-survey were analyzed using a paired samples t-test for each variable to determine significant changes. Participants who did not complete the post-survey were not included in *t*-test analyses. The hypothesis was that scores would change from the pre- to post-survey.

Responses were analyzed from 98 individuals who responded to post-survey interviews using a narrative approach to address the second aim. In the narrative approach, participants tell their stories to the researcher, and the researcher encourages the participants to expand upon their answers in search of additional meaning and detail about the environment and lived experiences (60). Analyses of interviews were conducted by a research team involving a graduate student and an advanced undergraduate student and supported by the PI. To analyze the interviews, researchers reviewed the interview guide and a selection of transcripts. From that initial review, each researcher wrote down key themes based on the research questions and compared lists with one another, which led to a developed list of codes. Next, each researcher coded the same five transcripts and compared codes. In instances of disagreement, differences were discussed until there was an agreed path for moving forward. After agreements were made, another five transcripts were reviewed. The remaining transcripts were divided and coded once an 80% agreement was achieved. Code categories were refined over time through literature review and upon review of quantitative analyses.

## 3 Results

### 3.1 Demographics

Demographic characteristics of participants are found in Table 1. Individuals were included if they completed a pre-survey and were assigned an iPad. Participants ages ranged from 55 to 100 with a mix of racial/ethnic identification. Most individuals’ primary language was English (77.7%) or Spanish (20.7%). Relationship status varied, with the highest group identifying as single; participants could choose more than one response. Most identified as retired, and about a quarter were unemployed. Most individuals lived alone and were lower income (less than $30,000 annually). Nearly half of the participants had a high school education or less, and half had some college or graduated college. Individuals self-reported health status, with the highest response being “*good*” health. Conclusively, over half (57.2%) reported having internet access, with 79.3% reporting never using a tablet before the pilot.

**Table 1:** Demographics of participants in 2021.

Characteristics		
	*N*	Mean (%)
Age	184	72.3
*Gender*		
Female	143	77.7
Male	41	22.3
*Racial/Ethnic group*		
White	104	56.5
Hispanic	25	21.7
Black	40	13.6
Native American/Alaska Native	9	4.9
Asian	2	1.1
*Primary language*	184	
English	143	77.7
Spanish	38	20.7
Other	3	1.6
*Relationship status (can choose multiple)*		
Single	64	
Divorced/separated	56	
Widowed	41	
Married/partnered	32	
*Current employment status*		
Retired	122	66.7
Unemployed	42	22.9
Employed	10	5.5
Other	9	4.9
Lives alone	130	70.7
*Income*		
Less than $30,000 annually	149	82.3
More than $30,000 annually	32	17.7
*Education*		
Did not complete high school	27	14.7
Completed high school/ GED	63	34.2
Some college	46	25.0
Graduated a 4-year college	39	21.2
Received graduate degree	9	4.9
*Self-reported health status*		
Poor	17	9.2
Fair	38	20.7
Good	74	40.2
Very good	38	20.7
Excellent	17	9.2

Data from all participants, including those who only participated in the pre-survey, are shown (*N* = 184). The numbers in this table reflect the number and percentage of participants who answered “yes” to this response.

### 3.2 Analysis

#### 3.2.1 Pre/post change

To address Aim 1, paired samples *t*-tests, shown in Table 2, showed statistically significant changes in participant scores pre- to post-survey for the QOL global measure, SI scale and subscales (social belonging and social connectedness), and the loneliness scale and emotional loneliness sub-scale, suggesting that participants had improved SI and loneliness following participation in eGen. Results were nonsignificant for the QOL scale and the social loneliness sub-scale.

**Table 2:** Paired samples *t*-test in social well-being outcomes.

Scale	Mean score		Cohen’s *d*
	Pre	Post	
Quality of life (QOL)	54.24	55.30	−0.13
QOL one item global measure	2.06	1.82**	0.28
Social isolation scale	23.28	24.32**	−0.25
Social connectedness sub-scal	11.3	11.79*	−0.15
Social belonging sub-scale	12.01	12.53	−0.21
Loneliness scale	2.44	2.16*	0.15
Emotional loneliness sub-scale	1.28	1.11**	0.18
Social loneliness sub-scale	1.16	1.05	0.08

Participants are included if they completed the pre- and post-survey (*n* = 145).

*p < 0.05; **p < 0.001.

#### 3.2.2 Qualitative findings

The second aim was analyzed using qualitative data from the post-survey interviews to understand how the eGen program contributed to improving participants’ social well-being. See Table 3 for themes, quotes, and numbers of comments/participants.

**Table 3:** Qualitative research questions by themes, quotes, and comment/participant frequency.

How did the program contribute to social well-being?		
Themes	Participant quotes	Comment/frequency
Enhanced mood & mental health	*It actually has impacted my mental well-being because like I said, with FaceTime, I have a friend, my best friend who lives far away, and I was able to see her for the first time in two years, that made me feel really good. –*Female, 69, White, non-Hispanic, English-speaking *It was just getting to be too much for me to always be in here, in the house. Stuck in the house, nowhere to go, nothing to do. When they introduced me to this program, it was the most wonderful thing that ever happened.* –Female, 71, White, non-Hispanic, English-speaking	61 comments by 52 participants
Increased quality of life	*For everything. I think it’s great that it keeps my mind going with this and getting to meet people and helping people out that I love. It’s something just to keep you active instead of just doing nothing…* -Female, 71, non-White, Hispanic, English-speaking *It made me a lot more independent; I can say that.* –Male, 66, Black, non-Hispanic, English-speaking *Yes, it has improved me. Finally I am doing better and better things for good nutrition, a better diet in order to improve my health.* *-* Male, 70, Hispanic, Spanish-speaking	72 comments by 52 participants
Offered sense of purpose & felt less alone	*It makes you feel like you are still a part of society…. To be able to set up a Zoom and be able to see your entire family and talk to everybody, it gives you a sense of being alive.* -Female, 80, Black, non-Hispanic, English-speaking *Because now when my sister or my nieces or nephews are all sitting around and we are all sitting around enjoying having a conversation about the phone or a tablet and stuff, I can join in now myself. I have somewhat of a say. I can join in.* *-*Male, 85, non-Hispanic, English-speaking	55 comments by 40 people
Improved use of video calling applications	*It really helped me connect more with my family members, especially my brother who is older than I am, and we FaceTime together now. We’re looking at library stuff to do books together, reading on the iPad so I’m happy. I could talk to my sister in Texas.* -Female, 63, White, non-Hispanic, English-speaking *Yes. I’ve done some Zoom events with my family and I’ve also done FaceTime with them and it’s been fun. One of my friends had a birthday and she had just moved and I did not have her address so I made a happy birthday video and I sent it to her on her Facebook. She called me, she was so thrilled that I had taken the time to make a little video for her birthday.* -Female, 74, White, non-Hispanic, English-speaking *Yes, it is an indispensable means of communication because I communicate with my whole family, I make video call groups. I even get my telemedicines or my medical appointments* via *Zoom.* *-*Female, 58, Hispanic, Spanish-speaking	29 comments by 27 people
Communicate more freely with people in their network	*I can communicate with my family better. I can communicate with my family that’s not here, better than just using the telephone. I can communicate with them more and in different ways, such as texting.* *-*Female, 65, Black, non-Hispanic, English-speaking *It’s helped because I can more easily email with both my family and my friends. I was using my iPhone before, and I had difficulty in using the little letters and numbers and it’s much easier. It’s much more accessible on the iPad.* -Female, 72, White, non-Hispanic, English-speaking *I connect with a grandson who lives in California, who I see when I talk to him. I learned that. And with one of my daughters who does not live near me, I send her messages, we talk and we see each other.* -Female, 75, Hispanic, Spanish-speaking	72 comments by 57 people

In addressing well-being and overall health, most older adults alluded that participating in eGen helped **enhance their mood and mental health (theme 1)**. Participants discussed feeling better about themselves and their situations after the pilot. They often described how effectively connecting with family and friends using their iPad has elevated their mood. Those who participated in virtual exercise classes or mindfulness activities stated that those activities made them feel better about themselves. A few participants noted that they surprisingly enjoyed joining classes and found them helpful. Participants also appreciated classes or activities that engaged them cognitively and felt they improved their memory and focus.

In addition to mood improvement, some individuals mentioned that their overall **quality of life (theme 2)** has improved after participating in the program. Participants described how they felt their minds were more active and engaged after gaining access to the iPad and the internet. Many participants also appreciated communicating better with others due to the technology, stating that this improved their lives meaningfully. Many people also felt good about learning how to use technology because they could now assist others who wanted to learn. This ability to “pay it forward” enabled people to feel good about receiving assistance from student mentors and making a meaningful contribution to others’ lives.

Further, eGen provided older adults with a **sense of purpose and feeling less alone (theme 3)**. Oftentimes participants mentioned feeling disconnected or helpless due to their age and the ever-changing world of technology. Participants stated that the iPad and eGen helped them find a renewed sense of purpose and social connection. In the past, they often felt out of touch or as if they could not contribute to the conversations of younger family members; however, now that they had a device and were learning to use it, participants gained a newfound ability to converse with others about interesting topics. In gaining this sense of purpose, participants started feeling less alone. One participant noted that connecting with others as they age becomes increasingly difficult but getting more involved with technology has helped with that challenge. By utilizing the iPad to talk or email, individuals felt they could use social media to keep up with and connect in new ways, such as posting a comment on someone’s picture.

One aspect of learning technology is the ability to communicate with others. Several participants commented how using **video calling applications to connect with others (theme 4)** increased their contact with loved ones. Participants felt they could more easily communicate with those who lived at a distance and found ways to participate in events using video calling platforms.

After participating in eGen, participants mentioned that it became easier to **communicate more freely with people in their network (theme 5)**. Mentors were able to teach participants multiple and effective ways to communicate with their friends and family through email, texting, or other message-type apps. Participants appreciated being able to communicate with others on their own (i.e., not having to rely on others for support) and communicated more after the pilot due to the iPad.

## 4 Discussion

As technology becomes more integrated into everyday life, ensuring digital inclusion for older adults is increasingly important due to the slower rate of technology adoption and usage among older adults compared to the overall population (61). The previous homogenous sample of program participants of mostly White individuals did not detect changes in SI, so we sought to expand eGen access to minority populations and those with lower income and education, as those groups are more severely impacted by digital exclusion than typical volunteer samples (62). The primary aims were to identify if an intergenerational technology program could contribute to social well-being for older participants in greater need of technological support and resources.

There are two key findings of the present research. First, results partially support the aim one hypothesis in that older adult participants’ scores significantly improved in overall social isolation, loneliness, and the global measure of QOL. Within subscales, program participants increased feelings of social connectedness (SI subscale) and decreased feelings of emotional loneliness. These types of findings are helpful in understanding the specific aspects of people’s social lives that may be influenced by the program. Increasing social connectedness is particularly important, as prior research shows it has a positive association with health and well-being in older adults (63). In this study, QOL (scale score) and social loneliness (loneliness subscale) did not significantly change before and after the program.

Secondly, qualitative results support aim two findings in that post-intervention interviews indicated that eGen met its goal of enhancing participants’ social well-being. Participants stated the program enhanced their mood due to improved connections with family, friends, and community programs, and they also talked about how the program made them feel like they had a renewed sense of purpose. Many found that using video calling applications (e.g., FaceTime and Zoom) enabled them to connect with others more regularly, and being able to more freely connect with people using technology through texting, emailing, and messaging helped people feel more integrated into society. The themes identified by this study provide further insight into the ways in which social well-being is impacted by the program. The novel contribution of this study is that addressing social well-being can occur through an intergenerational program that both teaches older adults about technology and utilizes technology to connect the generations.

According to researchers (64), technology can successfully contribute to older adults aging in place when the following conditions are met, needs and wishes are prioritized, technology is accepted, technology provides benefits, and when the technology is easy to use, affordable, and reliable. This program was designed to meet those needs and ensure inclusion was possible for all older adults, specifically those from disadvantaged communities. The pilot also aimed to ensure participants could connect with family, friends, and their community in new ways, as researchers (6) suggest a focus on technology training for social purposes. By incorporating extensive assistance around email, social media sites, and video calling, the participants in this intervention improved their social well-being by enhancing their mood, providing a sense of purpose, and offering new ways to connect with family/friends.

**Figure 1:**
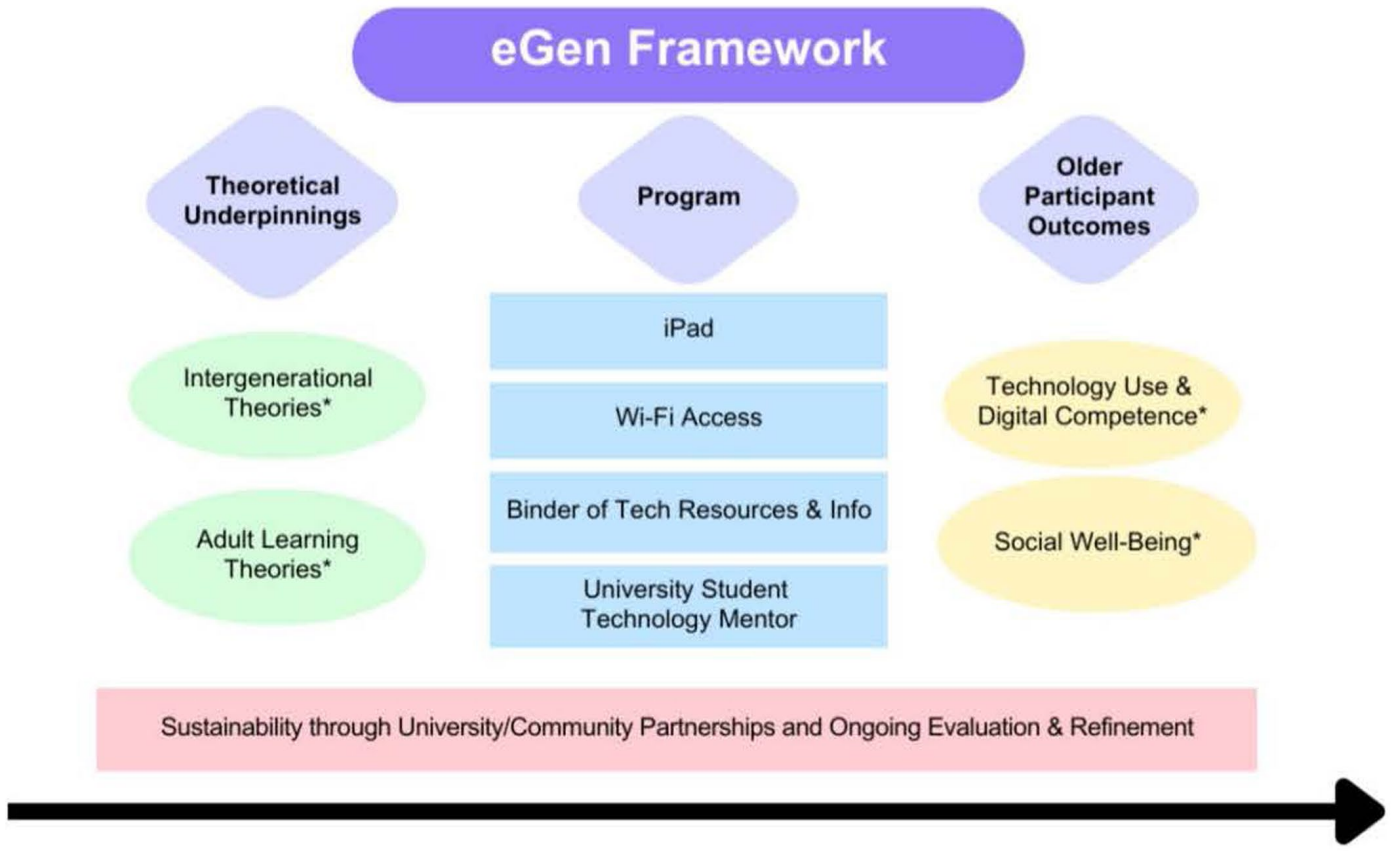
Engaging Generations (eGen) Framework. *Intergenerational theories include Social Exchange Theory and Contact Theory. Adult learning theories include the Adult Learning Theory and Sociocultural Learning Theory. Social Well-Being includes social isolation, loneliness, and quality of life.

The current study builds on the theoretical frameworks of implementation science (50) and introduces the Engaging Generations (eGen) Framework (shown in Figure 1). This theory is defined by five themes adapted toward intergenerational technology learning activities leading to higher technology usage for older adults and enhanced social well-being. This intervention included university student mentors, technology resources for older adults (inner setting), community partnership (outer setting), and the participants (individuals involved). Lastly, the pilot was accomplished, sustained, and successful with ongoing evaluations and refinement.

This pilot indicates that through program implementation, community/university partnerships can be effective and supportive (65). Due to eGen addressing a substantial community need (e.g., the need for digital inclusion and reduced SI among older adults) and having early success working with community partners, a larger number of individuals are interested in partnering than the program can support. This influx of participation indicates community/university partnerships can be successful. For more details on implementation strategies, see Supplementary material.

### 4.1 Limitations and future directions

This study has limitations. When interpreting the findings, it is important to note that this sample has no control group and did not use random sampling or random assignment. Furthermore, the data were collected in 2021 during the pandemic, when individuals may have gradually increased or resumed their everyday activities and, thus, may contribute to the analyzed responses. The pilot is ongoing and collecting continuous data to determine reproducibility in the current sample. Future research will examine the pathway between technology use and digital competence in social well-being to determine the magnitude in which technology use and learning drive the relationship toward better social well-being outcomes. Future research is needed to further understand the various social well-being outcomes. For example, QOL (scale score) and social loneliness scores did not significantly change from pre- to post-survey in this study, but qualitative results support that participants felt eGen contributed to enhanced quality of life and better health behaviors and outcomes. A larger sample would benefit from examining potential group differences in social well-being outcomes or relationships between social well-being outcomes. With larger sample sizes, we plan to investigate sub-samples, such as racial groups, gender, and income, to determine if there are significant changes from pre- to post-survey within sub-groups. Future research will also examine outcomes across intersectional groups (e.g., Black women who are widows, White men with little education) to further understand how intergenerational technology programs impact people differently. A strength of this sample is that 43.5% were from minoritized groups, and we continue to recruit older adults from underrepresented populations. Finally, we plan to investigate the impact of participation differences on changes in outcome measures as well as potential differences across the community sites since there was variation in support provided at each site.

A state-wide eGen program began in January 2022 and has gained continuous momentum, and we will continue to assess social well-being changes for participants. We believe that offering an iPad for completing pre- and post-surveys is an appropriate incentive for individuals to take part in the research and that phone surveys are an effective, sustainable method for collecting data to help avoid missing data issues. With low attrition rates, individuals are generally committed, and we are confident we have found the right balance of research participation, incentives, and program elements.

## 5 Conclusion

The current pilot study suggests that the eGen Program contributes to significant improvements in participants’ social isolation and loneliness, but further studies with bigger sample sizes are required to examine social well-being outcomes in relation to changes in technology use outcomes and investigate potential group differences in social isolation, loneliness, and quality of life. Further, qualitative findings revealed the program’s ability to foster new connections and strengthen existing social ties, ultimately contributing to improved social well-being for these individuals. These findings highlight the potential for technology and intergenerational programs to enhance older adults’ overall health and well-being.

## Data availability statement

The original contributions presented in the study are included in the article/Supplementary material, further inquiries can be directed to the corresponding author.

## Ethics statement

The studies involving humans were approved by The University of Rhode Island Institutional Review Board. The studies were conducted in accordance with the local legislation and institutional requirements. The participants provided verbal informed consent to participate in this study.

## Author contributions

CT: Formal analysis, Writing – original draft, Writing – review & editing, Data curation, Resources, Visualization. EP: Project administration, Writing – original draft, Data curation, Investigation. MB: Data curation, Formal analysis, Writing – review & editing, Resources. KS: Writing – review & editing, Project administration. SL: Methodology, Project administration, Writing – original draft, Writing – review & editing, Conceptualization, Funding acquisition, Supervision, Resources, Visualization.
