# Supplementary material for: Pilot testing the Engaging Generations (eGen) Program to address social well-being among lower-income older adults
Source: Front Public Health. 2024 Aug 9;12:1341713. doi: 10.3389/fpubh.2024.1341713 (PMC11341419; doi:10.3389/fpubh.2024.1341713)
Supplement: Supplementary file 1 [file Table_1.DOCX]

**Supplementary Material**

**Implementation Strategies**

We have learned that each community partner has different methods and processes for operating, and the community partners have learned that higher education has schedules, demands, and requirements that must be met. While these demands do not always align, we have learned that individuals involved in these community/university partnerships can remain motivated when they believe their organization and its participants or students will benefit in meaningful ways. When the partners work as a team to help participants, a program can be supportive of one another and thus successful in the long term. From a university standpoint, we learned there is a need for more investment in student support and supervision. From a community partner standpoint, we learned that participants appreciate when someone can easily answer questions or address concerns. Community partners helped with recruitment and connecting the university team with the older participants, and the university team worked to have a student available throughout the year. This ensured that participants could receive help and that URI eGen was sustained. This also helped the community partners keep the program and prevented it from being seen as a one-and-done initiative.
